# Understanding stable bi-female grouping in gibbons: feeding competition and reproductive success

**DOI:** 10.1186/s12983-015-0098-9

**Published:** 2015-03-05

**Authors:** Peng-Fei Fan, Thad Q Bartlett, Han-Lan Fei, Chang-Yong Ma, Wen Zhang

**Affiliations:** Institute of Himalaya Biodiversity Research, Dali University, Dali, 671003 Yunnan People’s Republic of China; Department of Anthropology, The University of Texas at San Antonio, San Antonio, TX 78249 USA

**Keywords:** Cao vit gibbon, Social system, Pair living, Socioecological model, Feeding competition, Reproductive success, Heterogeneity

## Abstract

**Introduction:**

Species of the order Primates are highly gregarious with most species living in permanent heterosexual social groups. According to theory in socioecology maximum social group size is limited by rates of intra-group feeding competition and associated increases in travel costs. Unlike other hylobatids, which are predominantly pair living, cao vit gibbons (*Nomascus nasutus*), and two other species of crested gibbon (*Nomascus* spp.) living in northern seasonal forest, regularly exhibit larger bi-female groups. To better understand the ability of northern gibbons to live in stable bi-female groups, we examined food distribution, feeding competition and reproductive success over a period of six years in a small cao vit gibbon population at Bangliang, Guangxi, China.

**Results:**

In general, we found weak evidences for within-group contest or scramble competition in our two study groups, which we attribute to high spatial and temporal heterogeneity in the distribution of their important food species. Nevertheless, the larger of the two groups studied increased feeding time and group spread during lean periods, factors that may limit cao vit gibbon group size to a maximum of two breeding females. Relative to tropical pair-living gibbons, there is no evidence that cao vit gibbons travel farther or spend more time feeding, but they did consume more leaves and buds and less fruit and figs. Despite their highly folivorous diet, the average inter-birth interval is comparable to tropical gibbon populations, and the survival rate of infants and juveniles in our study groups is high.

**Conclusion:**

Cao vit gibbons do not suffer obvious costs in terms of feeding competition and reproductive success by living in bi-female groups, but within-group feeding competition may determine the upper the limit of cao vit gibbon group size to a maximum of two breeding females. These findings contribute to a growing body of evidence that bi-female grouping can be a stable grouping pattern of gibbons in certain habitats and further emphasize the flexibility of gibbon social organization.

## Introduction

As an order, primates are highly gregarious with the overwhelming majority of species living in permanent heterosexual social groups. Consequently, it is assumed that group living carries with it a number of potential fitness advantages, including foraging efficiency [1], predator protection [2], cooperative resource defense [3], and protection from conspecifics [4]. Nevertheless, group size in primates varies considerably both within and between species highlighting the putative costs to group living. Chief among these is intra-group feeding competition, which will vary depending on the diet of the animals and distribution of resources in the environment [5]. Minimally, two modes of within-group feeding competition can be delineated: within-group contest (WGC) and within-group scramble (WGS) [6-8]. Contest (or interference) competition is characterized by agonistic interactions among group members, including contact aggression and displacement. Alternatively, scramble (or exploitation) competition occurs when animals lose access to resources (e.g., fruit) because those resources have already been exhausted by other group members. According to current socioecological theory, WGC should result if resources occur in discrete, high-quality patches that cannot feed all group members, while WGS should prevail if resources are of low quality, highly dispersed or very large. In WGC, net energy gain is dependent on rank with low-ranking females having a lower energy intake [8]. WGC may also result in differential energy intake among age-sex categories in small family groups [9]. Lower ranking animals may have to increase travel and feeding, or feed more on lower quality foods [8]. WGS is group-size dependent. As group size increases, net energy gain decreases; therefore, larger groups should deplete individual resources more quickly and animals will consequently adjust their foraging behavior by increasing travel, or by accepting a declining energy budget by feeding on lower quality foods [8]. Furthermore, scramble competition should increase when preferred resources are scarce. Finally, differences in net energy intake might translate into differential reproductive success of females [8,10], whereby females in larger groups experience reduced fertility.

Gibbons (Hylobatidae) are small arboreal apes that inhabit the rain forests of East and Southeast Asia. Though bi-male groups (*Hylobates lar*: [11,12]; *Symphalangus syndactylus*: [13]) and bi-female groups [14] have long been reported, members of this family typically live in groups comprised of one adult pair and one to three offspring. The apparent lack of flexibility in the social organization of gibbons relative to other primate families has led to speculation that the gibbon adaptive complex, characterized by monogamy and small-group territoriality, is evolutionarily constrained [15,16]. For example, Brockelman (2009: 211) has framed the question this way: “Have the gibbons’ marvelous adaptations to terminal branch feeding and frugivory…placed them in a specialized adaptive zone from which there is no evolutionary escape?” In light of this view, it is not surprising that discussions of gibbon feeding competition have focused on inter-group rather than intra-group feeding competition [16-18]. Significantly, the stable bi-female gibbon groups now well-documented in northern *Nomascus* gibbons [19-24] represent an alternative gibbon grouping pattern, emphasizing the flexibility of social organization in this family and inviting renewed attention to possible ecological constraints on group size in gibbons, and to the role of intra-group feeding competition and travel costs in particular.

The first reliable reports of gibbon groups with more than one female came out of work on western black crested gibbons (*N. concolor*) by Haimoff and colleagues [14] at Mt. Wuliang, China and was followed by observations on capped gibbons (*H. pileatus*: [25]), Hainan gibbons (*N. hainanus*:[19]), hoolock gibbons (*Hoolock hoolock*: [26]), lar gibbons (*H. lar*: [27]), and most recently, cao vit gibbons (*N. nasutus*: [24]). However, many of these bi-female groups were unstable [25-27]. With increasing field data from long-term studies (>6 years), researchers have demonstrated that the occurrence of bi-female groups in the three northern crested gibbons (*N. hainanus*; *N. concolor*; *N. nasutus*) are stable over many years and that co-resident females breed concurrently and repeatedly ([20-23], this study); engage in mutual grooming [21,28]; and maintain similar levels of proximity with adult males [22]. Most recently, Guang and colleagues [28] have demonstrated that females in bi-female groups actively cooperate in maintaining social relationships, rather than co-existing merely through tolerance or avoidance.

The addition of a second breeding female to a gibbon social group represents a significant increase in total group size. Because of the long juvenile period before dispersal in gibbons (>10 years: [12,29]), one adult female could potentially have up to four offspring living with her at once. Consequently, these bi-female groups have a greater number of individuals on average (*N. hainanus*: [20]; *N. concolor*: [21,23]; *N. nasutus*: [24]), up to a documented maximum of 9 group members (this study) and a theoretical maximum of up to 11. These offspring can represent equal competitors if they consume similar foods to adults [9]. Therefore, if intra-group feeding competition plays a role in limiting group size in gibbons we should expect increased competition in larger groups relative to smaller ones and, all things being equal, increased rates of intra-group feeding competition in socially polygynous gibbon groups relative to socially monogamous ones.

**Figure 1:**
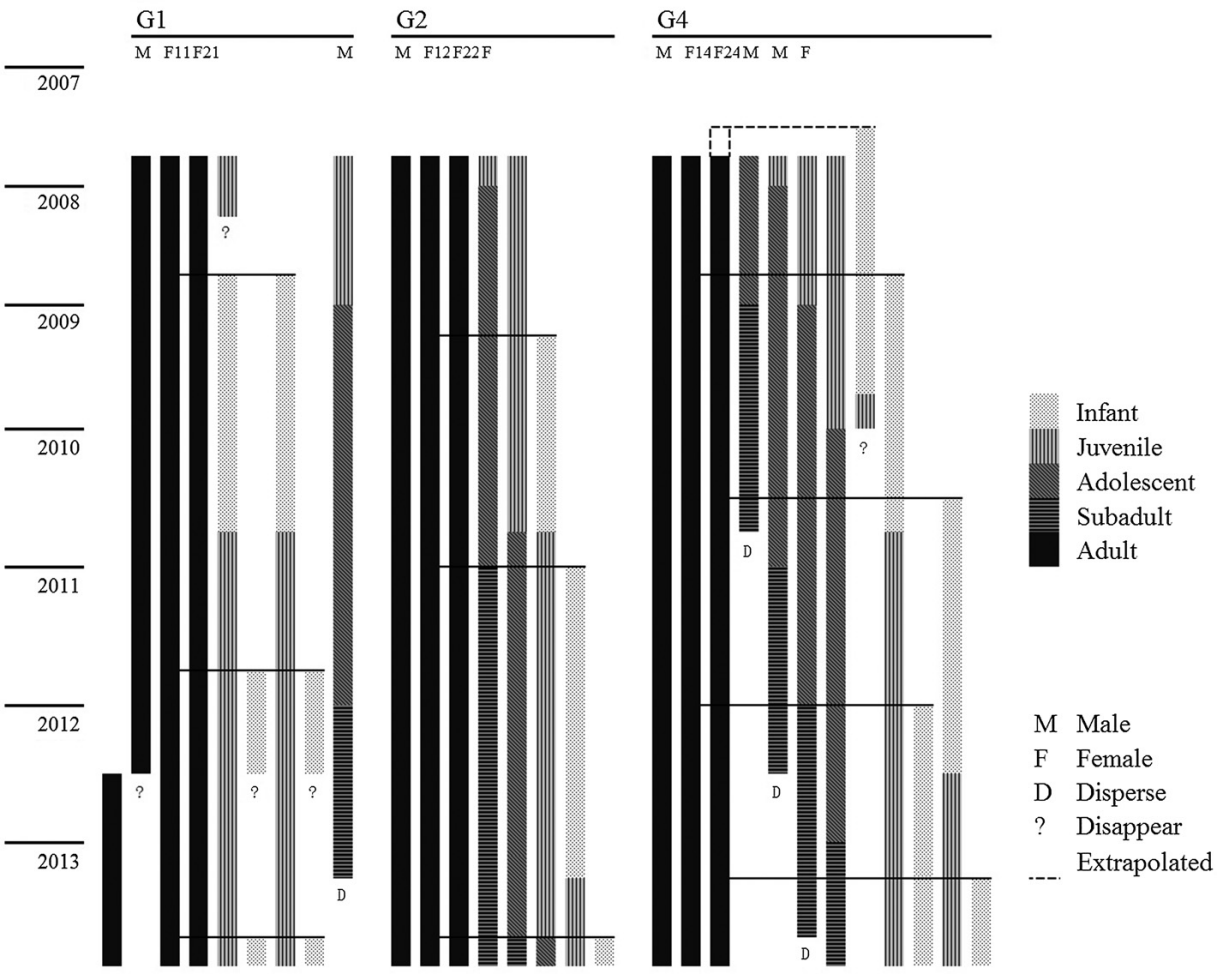
**Demography of three bi-female cao vit gibbon groups monitored between September 2007 and December 2013.** An infant born by F12 in February 2009 was thought to be independent at18 months old and F12 gave birth again in January 2011.

In this study, in order to better understand the relationship between feeding competition and group size in gibbons, we examined feeding competition, foraging behavior (diet, feeding time, group spread, travel time and distance), and reproductive success in three bi-female groups living in the Bangliang Gibbon Nature Reserve, Jingxi County, Guangxi, China (Figure 1). First, to document the role of WGC we investigated within-group behavioral differences to determine if there was a consistent pattern of age/sex class differences in diet, activity budget or food-related agonism. Second, to document the impact of WGS we (a) investigated temporal changes in behavior to determine if cao vit gibbons increased travel time and daily path length (DPL), spent more time feeding on low quality foods, or increased group spread during lean periods [8]; and (b) compared feeding behavior between two bi-female groups to see if the larger group suffered greater feeding competition in terms of increased travel, increased group spread, or more time feeding on low quality foods (e.g., buds and leaves). Third, given the hypothesized fitness consequences for females living in larger groups, we also report data on reproductive rates (i.e. inter-birth intervals) and infant and juvenile mortality in three bi-female groups in order to provide a basis for comparison to published data for females in pair-living groups elsewhere. Ideally, such comparisons would include groups with different numbers of females at a single site, but as there were no pair-living groups at our site this was not possible. Predictions tested in this study are summarized in Table 1. Finally, in addition to behavioral observations we also investigated food distribution patterns at the site in order to relate food distribution to different modes of feeding competition.

**Table 1:** **Predictions and related feeding competition modes used in this study**

**Level**	**Food competition mode**	**Predictions**	**Results G1 (small group)**	**G4 (large group)**
Within-group comparison	WGC	1. Age/sex differences in food-related agonism	Y	Y
		2. Age/sex differences in travel time.	Y	Y
		3. Age/sex differences in feeding time.	N	N
		4. Age/sex differences in diet (i.e. leaves & buds)	P	P
Seasonal variation	WGS	5. Groups travel further during lean period.	N	N
		6. Groups increase travel time during lean periods	N	N
		7. Groups increase feeding time during lean period.	N	Y
		8. Groups increase inter-individual distance and group spread during lean period.	N	Y
		9. Groups consume more leaves during lean period.	Y	Y
Between-group comparison	WGS	10. Larger group spends more time in travelling.		Y
		11. Larger group spends more time feeding.		N
		12. Larger group consumes more leaves.		N
		13. Larger group spreads wider than small group.		N
Between-site comparison	WGC and WGS	14. Decreased fertility in bi-female groups.		N
		15. Increased infant and juvenile mortality in bi-female groups		N

Y: yes, prediction met; N: no, prediction not met; P: prediction partly supported.

## Results

### Food distribution and fruiting phenology

Based on our previous work [30] we identified seven important food species, each of which contributed > 4.7% of the diet based on feeding time. During a subsequent research period between July 2012 and December 2013, one of us (CYM) determined that six of these seven species were among the top ten most consumed species by the same groups. Therefore, these species likely represent staple food species for cao vit gibbons and are more likely to play an important role in influencing feeding competition at this site. Of these, trees of *Ficus hookeriana*, *Choerospondias axillaris*, and *Spondias lakonensis* were rare (<4 trees/ha) at the site, while the remaining four species were more or less common (>8 trees/ha, Table 2). Five of the seven important food species did not show significant deviation from a Poisson distribution, which means these species are randomly distributed, while *Broussonetia papyrifera* and *Ficus glaberrima* showed a clumped distribution (Table 2). *Ficus glaberrima* and *Spondias lakonensis* were significantly taller and had larger DBHs than unimportant trees and *Ficus hookeriana* had a larger DBH than unimportant trees. Finally, *Burretiodendron hsienmu* trees were taller but had a smaller DBH on average than unimportant trees (Table 2).

**Table 2:** **Distribution pattern and characteristics of seven important food species in cao vit gibbon habitat, calculated from 73 20 × 20 m plots**

**Latin name**	**Contribution to the diet**	**Food part eaten**	**N of trees**	**N of plots**	**Density (trees/ha)**	**Tree height** ^**b**^	**DBH** ^**c**^	**CD index** ^**d**^
*Broussonetia papyrifera*	17.1	Bud, Fruit, Leaf	26	7	8.9	9.1 ± 1.5	14.8 ± 3.4	10.3**
*Ficus glaberrima*	13.0	Fruit, Bud, Leaf	61	26	20.9	11.7 ± 3.9**	26.3 ± 15.9**	3.1**
*Ficus hookeriana*	6.6	Fruit, Leaf, Bud	6	4	2.1	12.3 ± 5.1	33.7 ± 22.7**	1.6
*Tetrastigma pubinerve* ^*a*^	6.3	Fruit, Bud, Leaf	24	15	13.6			1.5
*Burretiodendron hsienmu*	5.5	Fruit, Leaf, Bud, Flower	34	16	11.6	9.6 ± 2.1*	14.1 ± 3.4*	2.9
*Choerospondias axillaris*	5.2	Fruit, Leaf	6	4	2.1	10.8 ± 2.6	24.9 ± 23.8	1.6
*Spondias lakonensis*	4.7	Fruit, Leaf	11	9	3.8	14.7 ± 7.5**	31.7 ± 23.8**	1.2
All other trees			1072	73	367.1	8.9 ± 2.7	16.7 ± 7.2	

^a^Height and DBH are not available for *Tetrastigma pubinerve,* which is a liana species.

^b^and ^c^For statistical analysis height and DBH were compared to all other trees (* P < 0.05; ** P < 0.01).

^d^If the distribution pattern of the species significantly deviates from Poisson distribution (** P < 0.01).

**Figure 2:**
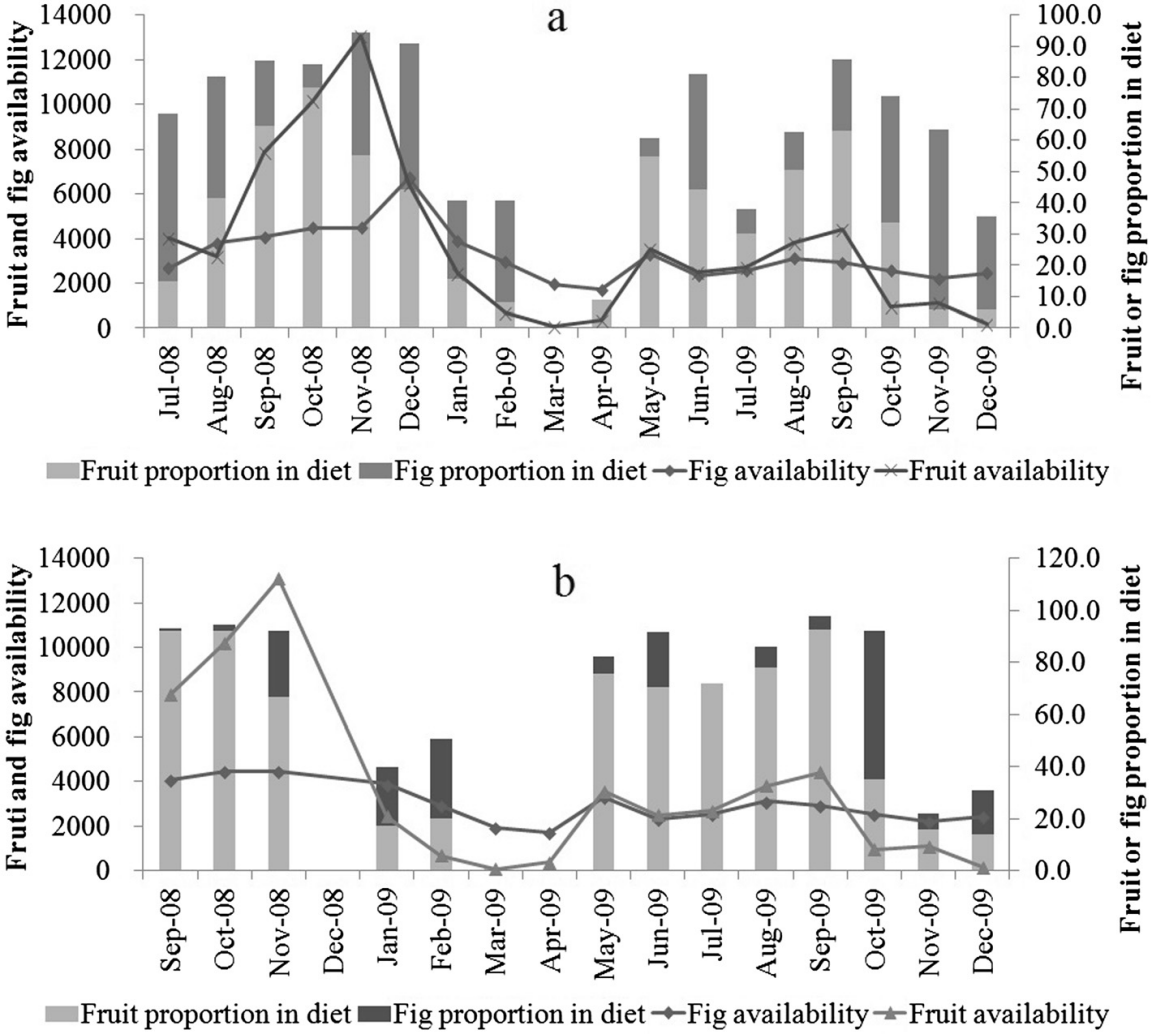
**Seasonal variation of fruit and fig availability and fruit and fig proportion in the monthly diet of G1 (1a) and G4 (1b).**

The production of ripe fruit at the study site exhibited extreme monthly and annual variability. Fruit was more abundant between July and December in 2008 than in 2009, probably as a result of less rainfall in 2009. *Tetrastigma pubinerve,* an important food species for the gibbons [30], produced a lot of fruit from August to December in 2008. However, this species produced almost no fruit in 2009. Based on the food availability index we were able to distinguish two periods of marked fruit scarcity, or lean periods. The first ranged from February to April 2009 and the second occurred from October to December 2009. During each of these months (N = 6) the fruit availability index was below 1,200. By contrast, in all other months (N = 12) the availability index was at least 2,400 (Figure 2). In the analysis below these months are referred to collectively as the fruiting period.

### Food-related agonism and age/sex differences in diet and activity budget

Both food-related agonistic interactions and displacements were rare in our two study groups (Table 3). In both groups, juveniles received far more food-related agonism from adults than did other age classes (10 of 11 instances). In G1, the juvenile spent more time travelling than the other three members (Wilcoxon matched pairs test: juvenile – male1, Z = −2.9, P = 0.003; juvenile – F11, Z = −2.6, P = 0.010; juvenile – F21, Z = −3.2, P = 0.001; N = 18 in all tests). But the male1 spent less time feeding than all other member (Wilcoxon matched pairs test: male1– F11, Z = −3.7, P < 0.001; male1 – F21, Z = −3.3, P = 0.001; male1 – juvenile, Z = −2.3, P = 0.019; N = 18 in all tests). F11 spent more time travelling (Wilcoxon matched pairs test: Z = −2.2, P = 0.028; N = 18) and feeding (Wilcoxon matched pairs test: Z = −2.5, P = 0.012; N = 18) than F21, probably due to F11 spending more time searching for insects than F21 (Wilcoxon matched pairs test: Z = −2.0, P = 0.044; N = 18). In general, group members shared a similar diet except that the male1 consumed more fruit (Wilcoxon matched pairs test: male1– F11, Z = −2.8, P = 0.005; male1 – F21, Z = −2.8, P = 0.005; male1-juvenile, Z = −1.9, P = 0.053; N = 18 in all tests) and less insects than other members (Wilcoxon matched pairs test: male1– F11, Z = −3.2, P < 0.001; male1 – F21, Z = −2.8, P = 0.006; male1-juvenile, Z = −2.9, P = 0.003; N = 18 in all tests). No difference was found between the two females except that F11 spent more time searching for insects.

**Table 3:** **Food-related agonistic interactions or displacements in group G1 (1,175 h observed from July 2008 and December 2009) and G4 (776 h observed from September 2008 and December 2009)**

**Group**	**Initiator**	**Receiver**	**N**
G1	Adult male	Juvenile	2
	F21	Adult male	1
G4	Adult male	Juvenile	3
	F14	Juvenile	2
	F14	Adolescent	2
	Adult male	Adolescent	1

Except for one case from G1, all agonistic interactions were observed during lean periods between February and April or October and December, 2009.

**Figure 3:**
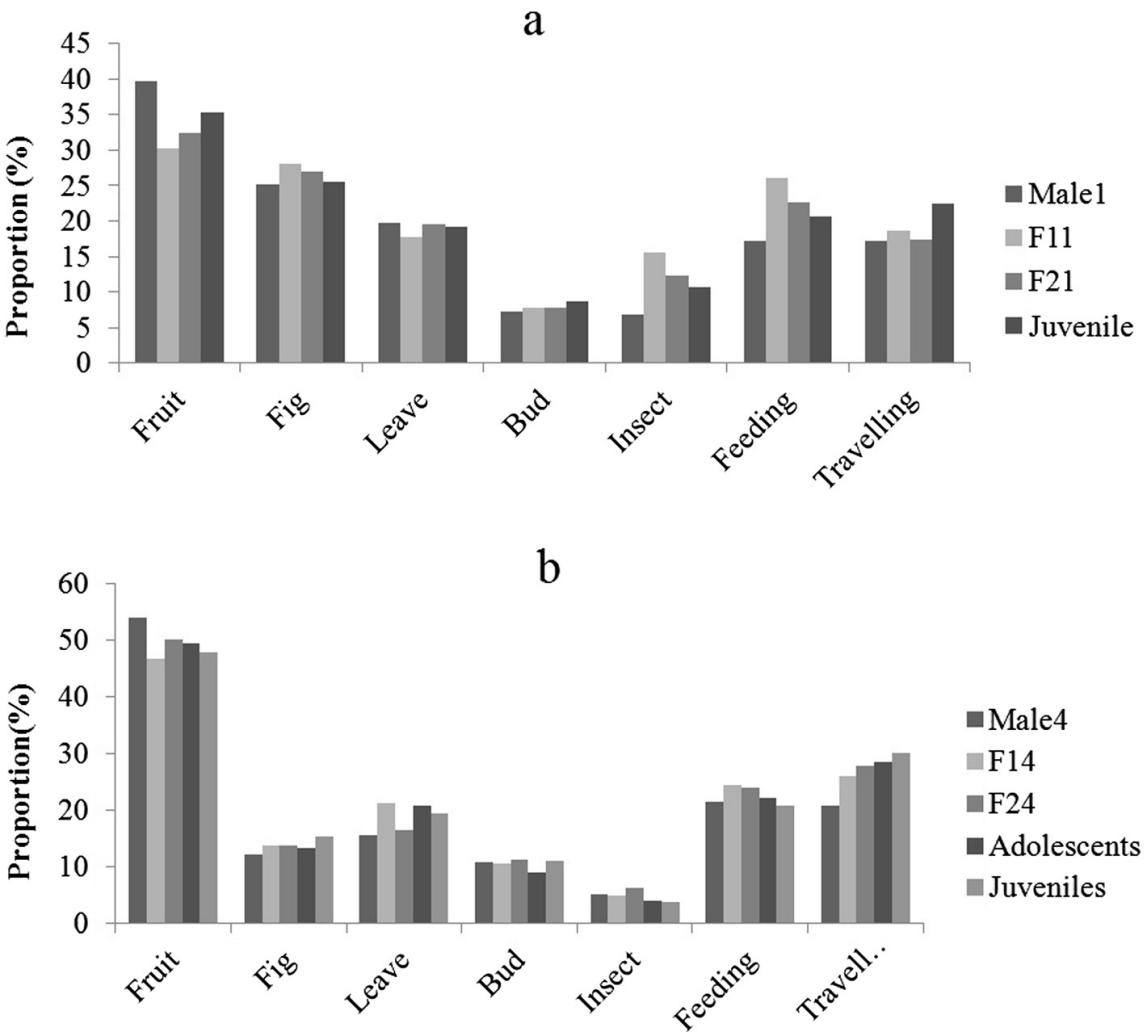
**Diet and time budget differences among members in G1 (2a) and G4 (2b).** Data for the two adolescents were pooled as were data for the two juveniles.

We found a similar pattern in G4. No differences were found in diet or activity budget between the two adult females. Both juveniles and adolescents spent more time in travelling than the adult male and more than the adult females in G4 (Wilcoxon matched pairs test: male4 – juveniles, Z = −3.4, P = 0.001; male4 – adolescents, Z = −3.3, P = 0.001; F14 – juveniles, Z = −2.7, P = 0.007; F14 – adolescents, Z = −2.5, P = 0.012; N = 15 in all tests). Juveniles spent less time feeding than females (Wilcoxon matched pairs test: juveniles – F14, Z = −2.5, P = 0.013; juveniles – F24, Z = −2.8, P = 0.006; N = 15 in all tests). Juveniles ate less fruit (Wilcoxon matched pairs test: Z = −2.2, P = 0.031; N = 15) but more leaves (Wilcoxon matched pairs test: Z = −2.3, P = 0.021; N = 15) and figs (Wilcoxon matched pairs test: Z = −2.0, P = 0.05; N = 15) than the adult male. Adolescents spent less time searching for insects than one female (Wilcoxon matched pairs test: F24 - adolescents: Z = −2.2, P = 0.026; N = 15) (Figure 3).

### Temporal variation in foraging behavior in response to fruit scarcity

The proportion of fruit and figs in the diet varied from 0.9% in March 2009 to 94.3% in November 2008 in G1 (Figure 2a) and from 0.6% in April 2009 to 97.8% in September 2009 in G4 (Figure 2b). When fruit was less available, both groups fed more on leaves and buds [31]. Contrary to our prediction, neither group spent more time travelling during the lean season (Mann–Whitney *U* test: G1: Z = −1.8, P = 0.075, N_fruit month_ = 12, N_lean month_ = 6; G4: Z = −1.2, P = 0.239, N_fruit month_ = 9, N_lean month_ = 6). On the contrary, both groups decreased DPL during lean periods (G1: lean periods: 890 ± 323 m, range: 354–1538 m, N = 20; fruiting periods: 1440 ± 454 m, range: 494–2361 m, N = 39; Mann–Whitney *U* test: Z = −4.2, P < 0.001; G4: lean periods: 920 ± 403 m, range: 475–1983 m, N = 14; fruiting periods: 1471 ± 502 m, range: 582–2282 m, N = 16; Mann–Whitney *U* test: Z = −2.9, P = 0.004). G1 did not spend more time in feeding (Mann–Whitney Utest: Z = −1.5, P = 0.134, N_fruit month_ = 12, N_lean month_ = 6) and did not increase inter-individual distance (Mann–Whitney *U* test: Z = −0.6; P = 0.542, N_fruit month_ = 12, N_lean month_ = 6) or group spread (Mann–Whitney Utest: Z = −0.7, P = 0.512, N_fruit month_ = 12, N_lean month_ = 6) in lean periods (Table 4). On the other hand, G4 increased feeding time (Mann–Whitney *U* test:Z = −2.8, P =0.006, N_fruit month_ = 9, N_lean month_ = 6), inter-individual distance (Mann–Whitney *U* test: Z = −2.4, P = 0.018, N_fruit month_ = 9, N_lean month_ = 6), and group spread (Mann–Whitney *U* test:Z = −2.4, P = 0.018, N_fruit month_ = 9, N_lean month_ = 6) in lean periods (Table 4). Except for one case in G1, all food-related agonism was observed during lean periods (Table 3).

**Table 4:** **Mean inter-individual distance and group spread (represented as maximum inter-individual distance) in two bi-female cao vit gibbon groups in China**

	**G1**			**G4**		
	**N**	**Mean inter-individual distance**	**Group spread**	**N**	**Mean inter-individual distance**	**Group spread**
Jul-08	25	11.0	16.3			
Aug-08	273	7.4	11.7			
Sep-08	221	7.6	11.8	68	4.6	7.4
Oct-08	196	8.6	13.2	121	4.3	7.4
Nov-08	175	16.8	26.2	103	4.0	7.3
Dec-08	202	11.6	17.4			
Jan-09	118	9.8	14.8	116	4.4	7.5
**Feb-09**	192	15.0	23.5	239	9.4	15.9
**Mar-09**	301	11.5	18.3	300	7.8	13.0
**Apr-09**	133	8.6	13.3	209	5.7	9.2
May-09	84	6.8	11.2	198	8.6	13.9
Jun-09	142	15.8	25.5	344	7.3	12.1
Jul-09	126	5.7	9.5	153	6.2	10.5
Aug-09	97	6.3	11.2	313	5.1	8.5
Sep-09	201	8.6	15.3	221	4.8	8.0
**Oct-09**	132	5.7	9.6	162	5.9	9.3
**Nov-09**	98	8.9	13.8	106	11.4	18.7
**Dec-09**	177	13.9	22.0	223	9.0	14.3
Lean period		10.6 ± 3.5	16.8 ± 5.4		8.2 ± 2.2	13.4 ± 3.7
Fruiting period		9.7 ± 3.6	15.3 ± 5.4		5.5 ± 1.6	9.2 ± 2.4
Z		−0.610	−0.656		−2.357	−2.359
P		0.542	0.512		0.018	0.018

Months in bold was classified as fruit scarce based on the mean value of fruit production in 2009.

### Differences between groups

Adult members in both groups spent similar amounts of time feeding, but on average individuals in the larger group (G4) spent significantly more time travelling and less time resting than in the smaller group (G1, Table 5). However, G4 did not travel farther than G1 (G1: mean 1182 ± 474 m, range 354 – 2361 m, N = 50; G4: mean 1214 m ± 531, range 475 – 2282, N = 30; Mann–Whitney *U* test: Z = − 0.1, P = 0.881). Individuals in G4 ate more fruit and fewer figs than individuals in G1 (Table 5), but if we combine fruit and figs into a single category there is no significant difference between these two groups, though G1 consumed more insects than G4 (Table 5). No differences were found in the consumption of leaves or leaf buds between groups. Wilcoxon matched pairs test showed the larger group (G4) maintained closer inter-individual distance than the smaller group (G1: Z = −2.7, P = 0.006, N = 15) using three sample distances.

**Table 5:** **Comparison in the time budget and diet of the adults in gibbon groups G1 and G4**

	**G1**	**G4**	**Wilcoxon matched pairs test**	
**Activities**	**Mean ± SD (%)**	**Mean ± SD (%)**	**Z**	**P**
Feeding	22.0 ± 5.5	23.2 ± 8.3	−0.6	0.57
Travelling	17.4 ± 6.2	24.7 ± 8.5	−3.2	0.001
Resting	45.0 ± 7.6	30.0 ± 10.2	−3.2	0.001
Social	14.8 ± 8.2	20.3 ± 9.5	−3.1	0.002
Fruit	34.4 ± 25.2	50.2 ± 34.8	−3.1	0.002
Fig	23.1 ± 17.9	13.1 ± 15.1	−2.7	0.007
Fruit + Fig	57.5 ± 28.5	63.2 ± 35.1	−1.8	0.078
Leaves	20.8 ± 23.2	17.6 ± 25.2	−1.0	0.311
Buds	8.9 ± 20.2	10.7 ± 24.8	−0.1	0.925
Insects	11.6 ± 16.2	5.3 ± 9.0	−2.8	0.005

### Demography and female reproduction

At the beginning of this study G1 consisted of one adult male, two adult females and two juveniles. One juvenile disappeared in February 2008, F11 gave birth in November 2008, and F21 gave birth in December 2008. Both females again gave birth in November 2011. The original adult male of G1 was replaced by a new male in July 2012. Coincident with a period of male replacement, two infants born in 2011 disappeared and are presumed to have died. F11 gave birth in December 2013 and F21 in November 2013. The juvenile, by then a sub-adult male, dispersed in April 2013. The group consisted of seven individuals in December 2013 (Figure 1).

G2 consisted of one adult male, two adult females, one adolescent, and one juvenile in December 2007 and increased to eight individuals by the end of the study due to three births by F21 (Figure 1). The other female, F22 in G2, never reproduced during the six years of the study. This female was thought to be too old to give birth because her pelage became darker over the course of the study, a phenomenon characteristic of aging females among *Nomascus* gibbons [32].

G4 consisted of eight individuals including one adult male, two adult females, two adolescents, two juveniles, and one infant in December 2007 and increased to nine due to a birth by F14 in November 2008. A small juvenile disappeared in November 2009 just after it became fully independent at 3 years old (Figure 1). F14 gave birth to another infant in January 2012. And F24 in G4 gave birth in July 2010 and again in March 2013. The group size of G4 remained at least eight individuals and occasionally nine individuals during the six-year observation period, representing the largest group in the population. It is possible that nine individuals is the maximum number of animals this group can accommodate. In 3 of the 4 cases, a subadult individual dispersed within 1–5 months of the birth of a new infant (Figure 1).

Eight floating males (black phase) and nine floating females were also observed during the six years. Co-dispersal of three males in one case and two females in another case was observed. The three males were observed on six days between February and November 2009. They finally established their own territory in the site and attracted a female to live with them [24]. The co-dispersing females were observed on four days between August 14 and September 17. They fed on the same tree, groomed each other, and always remained within 20 m of each other. Subadult males produced solo bouts before dispersal but subadult females only produced great calls with resident adult females and never sang solo bouts before dispersal. Floating individuals did not call.

In summary, five of the six adult females in our three study groups reproduced at least twice between December 2007 and 2013. One old female in G2 never gave birth during the study. Two infants in G1 died during male replacement and one juvenile each died in G1 and G4. All other offspring survived to dispersal at about 10 years or to the end of the study. The inter-birth interval (IBI) was 31.0 ± 6.6 months (range: 23–38 months, N = 8) and 33.5 ± 5.5 months if we include only surviving infants (range: 23 – 38 months, N =6). Although the sample size is small, IBI does not appear to differ between groups (G1 mean = 30.3, N = 4; G2 mean = 29.0, N = 2; G4 mean = 35.3, N = 2). Group size varied between five and nine individuals due to births, disappearances/deaths, and dispersals and was always larger, on average, than in pair living groups of other species (Figure 1).

## Discussion

We studied feeding competition and female reproductive success in a cao vit gibbon population dominated by bi-female groups (14 of 17 groups: Le TD, Fan PF, Yan L, Le HO, Josh K: The global cao vit gibbon (*Nomascus nasutus*) population. unpublished). The sample size was limited because the population straddles the international border between China and Vietnam. Nevertheless, we successfully monitored group dynamics of the three bi-female groups that range wholely or partly on the China side of the boarder beginning in December 2007 and documented patterns of feeding and ranging in two of these groups between January 2008 and December 2009. Due to the difficult karst terrain, we could not follow gibbons at our site. Instead, we observed them from 50–500 m away from a series of observation posts. Results reported here demonstrate the feasibility of these methods for collecting basic ecological and demographic data on arboreal primates in karst forest ([33-35], this study), as well as the potential for these methods to be used to observe arboreal primates in other difficult terrian where trees are small.

### Feeding competition and characteristics of important food species

Exploitation of large feeding patches predicts WGS and weak WGC, which is consistent with our observations (see below). The important food species for cao vit gibbons either exist as large trees or grow in large clumps. Full grown trees of *Ficus glaberrima, Ficus hookeriana,* and *Spondias lakonensis* are the largest trees in this habitat. Foraging gibbons could visit a single fruiting tree of these species repeatedly over several days (PFF personal observation). *Broussonetia papyrifera* was similar to trees of non-important species in terms of height and DBH, but this species usually occurs in clusters around abandoned fire pits used for charcoal production and several trees with continuous tree crowns can serve as a single large feeding patch for gibbons.

### Feeding competition within bi-female gibbon groups

#### Contest competition

We found evidence of only weak WGC in terms of food-related agonism or age/sex-related differences in diet in our two study groups. In general, increasing numbers of group members should increase instances of agonism [36], but contrary to this pattern we observed only 11 agonistic episodes during feeding in over 2,000 hour of observation during 300 days (0.04 cases/d), which was lower than that observed in another bi-female group of western black crested gibbon (0.3 cases/d: [22]), in pair living siamangs (1.5 cases/d: [37]), or in lar gibbons (0.08 cases/d: [18]). Given the observation distance (50–500 m), we might have overlooked some subtle agonistic behaviors, such as open-mouth threats that have been observed in western black crested gibbons [22], but frequent grooming and social play among members in these groups (11.3% and 16.9% of active time for G1 and G4 [31]) suggests that social relationships were in fact harmonious. It is important to note that no food-related agonism was observed between adult females living in the same group. Both females groomed each other and also groomed the adult male and received grooming from him (Fan et al., in preparation). Although we could not reliably identify individual juveniles in each scan, we did observe females grooming both similar-sized juveniles who were mothered by different females. In general, group members shared similar diets and no differences in diet were detected between females living in the same group.

In both groups, juveniles consumed less fruit and spent more time travelling than the resident adult male (Figure 2) and in all but one case juveniles were the targets of intra-group agonism (Table 3). These findings might indicate that juveniles were the recipients of contest competition, although other factors may account for the observed differences in activity budget. For example, young gibbons may have different nutritional goals or may travel more as a part of social (e.g., chase) or solitary (e.g., exploration) play [18]. However, because we did not distinguish directed movement between trees (i.e., travel) from movement within trees (i.e. move) during data collection, it is not possible for us to determine precisely what accounts for the observed differences in activity.

#### Scramble competition

We found some evidences of WGS within this population. With group size ranging from 8–9 individuals, group G4 is the largest gibbon group ever recorded in the wild. Adult members of G4 spent more time travelling on average and less time resting than adults of G1. However, greater travel time did not translate into longer DPL. It is possible that gibbons in G4 had to spend more time foraging (slow searching movement) between patches, or had to accommodate the slower movements of juveniles in this group.

Contrary to predictions, neither group increased DPL in response to fruit scarcity. Instead, they altered their diet, consuming more leaves and buds [31]. We also found that G4 altered its behavior in response to fruit scarcity in other ways. G4 increased feeding time and group spread during lean periods, which may represent a strategy to avoid food competition in lean seasons. All cases of agonism in G4 also occurred during lean periods. Together these results suggest that this large group experienced increased feeding competition in lean periods, which may impose an upper limit on group size. Subadults in G4 almost always dispersed soon after the births of infants, which might mean that G4 approached its maximum group size at nine individuals.

### Stable bi-female groups in cao vit gibbons

It has been argued that the benefits of living in permanent groups include enhanced predator avoidance, foraging advantages, and avoidance of conspecific threat (e.g., infanticide), while the main cost of living in groups is within group feeding competition [5], which might result in increased travel costs or a switch to a lower quality diet. A preliminary comparison offers no evidence that cao vit gibbons travel farther than tropical gibbons living in adult pairs (on average 1.2 km, N = 16, Table 17.2 in [38]). On the other hand, relative to other gibbons, rates of folivory among cao vit gibbons are high. Though in this case, the lower proportion of fruit and figs in the overall diet was primarily a result of fruit scarcity during lean periods, not by within group food competition. Our study groups consumed > 90% fruit (including figs) in their monthly diet during fruiting periods, which is comparable or even higher than monthly fruit and fig consumption in tropical gibbons [18,37,39-41]. However, in contrast to other gibbon genera, cao vit gibbons switched their diet and mainly depended on more abundant leaves and buds in lean periods when the proportion of fruit and figs in the diet decreased to < 1%. As a result, their annual diet was dominated by leaves (G1: 49.1%; G4: 54.9% [31]). In other words, it is highly likely that a small pair-living group at the site would have a similar overall diet to bi-female groups. Nevertheless, if leaves do represent low quality resources for cao vit gibbons, one might assume that gibbons in these bi-female groups would be subject to reproductive costs. However, there is no evidence that their highly folivorous diet translates into lower reproductive success. With the exception of a single old female, the adult females in the three groups we studied bred repeatedly. IBIs of breeding females in our groups and other bi-female groups, for which there are data, were comparable or even shorter than the IBIs reported for tropical pair-living groups (Table 6 [20,23,42-46]). Except for two infants that died during a male replacement incident, all other infants (N = 8) survived to independence at 3 years old. Similarly, only two small juveniles died over the 7-year study period.

**Table 6:** **Inter-birth intervals of wild gibbon populations**

**Species**	**Inter-birth interval (month)**	**Range (month)**	**N**	**Grouping pattern**	**Literature**
*Hylobates lar*	41 ± 9.1	34-71	17	Pair, Bi-male	[42]
	120		1	Pair	[43]
	26.5	22-31	2	Pair	[44]
*H. agilis*	38.4 ± 4.8		5	Pair	[45]
*Hoolock leuconedys*	49		1	Pair	Fan PF, unpublished data
*Symphalangus syndactylus*	33.7 ± 3.5	28.5-38.1	5	Pair, Bi-male	[46]
	60	48-72	2	Pair	[43]
	>36		3	Pair	[44]
*Nomascus hainanus* ^*1*^	24 ± 1.6	22-26	4	Bi-female	[20]
*N. concolor*	42.3 ± 9.2	37-53	3	Bi-female	[23]
*N. nasutus*	31 ± 6.6	23-38	8	Bi-female	Present study

^1^Calculated from Table II in [20]. Only birth dates of consecutive infants known to within a month were used to calculate the inter-birth interval.

Though preliminary, our finding that the reproductive success of cao vit gibbons does not differ appreciably from that of southern populations requires further consideration, particularly in light of the extreme seasonality of their habitat and clear differences in diet. One possibility is that cao vit gibbons may have an evolved capacity to obtain sufficient nutrition from leaves and buds necessary to tolerate lean periods, a view that is consistent with larger body size in *Nomascus* relative to gibbons of the best studied genus *Hylobates* [47]. It is also possible that cao vit gibbons, like some other primate species [48,49], may have the ability to deposit fat reserves during periods when fruit is abundant to help them cope with fruit scarcity.

In any case, if cao vit gibbons do not suffer greater reproductive costs from living in bi-female groups, then any benefits of living in larger groups—foraging efficiency, predation protection, range defense, or protection from conspecifics—should promote multi-female groups. Given that predation on gibbons is extremely rare, predation avoidance may not represent a significant benefit for females living in bi-female groups relative to those living in pairs. On the other hand, resident females in the same group always produced similar-aged juveniles. In instances where these similar-aged offspring are also females they may subsequently disperse together, which might increase survival during dispersal and increase the probability of establishing their own territory ([24], this study). In addition, co-resident females might benefit from improved inclusive fitness if they are kin, which is possible if females born in the same group disperse together (this study). If so, then an unpaired female may benefit more by joining an established adult pair than by waiting to join an unmated male. But, even if females do not obtain any direct or indirect benefits, bi-female groups could form if the cost of evicting the second female exceeds the cost of tolerance [22,50].

## Conclusion

The data presented here, together with observations of other northern *Nomascus* populations ([20-24,28], this study), suggest that bi-female grouping is a stable grouping pattern for gibbons in certain habitats. Given the limited sample size of our study, any conclusions about the mechanisms favoring bi-female grouping in *Nomascus* gibbons must be tentative. Nevertheless, we have attempted to show how intra-group feeding competition, combined with high spatial and temporal heterogeneity in resource abundance may help us understand why bi-female groups represent the dominant grouping pattern in this cao vit gibbon population. Feeding competition in this population is rare likely owing to high spatial and temporal heterogeneity of resources, including leaves. However, when evidence of WGC and WGS was detected it was in the larger group during lean periods. This combined with the timing of subadult dispersal suggests that group size is capped at two adult females. In order to better understand feeding competition and its role in determining gibbon social organization, detailed comparative research of food distribution, feeding party size, feeding bout length, and seasonal variation of feeding behavior in relation to food availability using standardized methods will be extremely useful. Only in this way will we be able to discern if the ability to form stable multi-female groups is unique to gibbons of the genus *Nomascus*, or if it is merely another example of the social flexibility of all gibbons, and to some extent all primates.

## Methods

### Ethical approval

All observations were conducted in Bangliang Gibbon Nature Reserve under the permission form the Guangxi Forestry Bureau and the Management Bureau of Bangliang Gibbon Nature Reserve. No any gibbons were handled during the research. All research adhered to the legal requirements of China and the American Society of Primatologists (ASP) Principles for the Ethical Treatment of Non-Human Primates (https://www.asp.org/society/resolutions/EthicalTreatmentOfNonHumanPrimates.cfm).

### Study area

We carried out the study in Bangliang Gibbon Nature Reserve (486 to 926 m above sea level, 22°55′N, 106°29-30′E, established in 2009), Jingxi County, Guangxi, China, which is continuous with the Cao Vit Gibbon Conservation Area in Trung Khanh, Cao Bang, Vietnam (established in 2007) [51]. This karst limestone area is characterised by steep-sloped sharp-peaked mountains surrounded by two branches of the Quay Son River flowing into Vietnam. The forest is monsoon tropical forest but was degraded by selective logging, fuelwood collection, charcoal making, and agriculture before the nature reserves were established [30]. The total area of good forest available for gibbons is estimated to be 2,200 ha [51]. The climate is humid tropical and shows obvious seasonal variations in temperature and rainfall. The annual mean temperatures is *c*. 20.0°C and monthly mean temperatures range from 9.8°C in the coldest season to 26.6°C during the warmest season. Annual precipitation was 1,804 mm in 2008 and 1,363 mm in 2009 [17]. Additional information on the study area has been published elsewhere [30,31,51].

### Gibbon population and study groups

This population was rediscovered in Vietnam in 2002 [52] and in China in 2006 [53] and represents the only known population of cao vit gibbons. Since the discovery of this population, surveys have shown that bi-female groups predominate (14 of 17 groups Le TD, Fan PF, Yan L, Le HO, Josh K: The global cao vit gibbon (*Nomascus nasutus*) population. unpublished; 2 of 3 groups [54]; 3 of 5 groups *Geissmann* T, La QT, Trinh DH, Dang NC, Pham DT, Vu DT: Report on an overall survey of the cao vit gibbon population Nomascus sp. cf. nasutus in Trung Khanh District, Cao Bang Province (second overall survey). unpublished). Three bi-female groups range wholely or partly in China and we started to monitor the dynamics of all three groups monthly beginning in December 2007 [24]. Non-adult gibbons were classified into 4 categories: infant (0–2 years of age), juvenile (2–5 years of age), adolescent (5–8 years of age), and subadult (>8 years of age) [18,29].

G1, one of the smaller groups in the population at the beginning of the study, consisted of one adult male, two adult females, and one juvenile in August 2008. It increased to six individuals due to one birth each in November and December 2009 and then to seven individuals by the end of December 2013. Alternatively, G4 represents the largest documented social group in any gibbon population, consisting of eight individuals (one adult male, two adult females, two adolescents, two juveniles and one infant) at the beginning of the study in August 2008 and increasing to nine individuals due to a birth in November 2008. At the beginning of data collection G1 had 4 individuals and group G4 had 8, while during most observation months G1 had 6 individuals and G4 had 9. It is our contention that a difference in group size of 50-100% is biologically meaningful and should be associated with detectable differences in foraging behavior. The third group, G2 ranged from five (one adult male, two adult females, and two juveniles) to eight animals over the course of the study (September 2007 to December 2013). Its range extended across the international boarder so while we monitored demographic changes in this group, systematic data on feeding and range use was limited.

We could reliably distinguish the two females (F11 and F21) in G1 based on their white face ring and crest shape only after June 2008. It was easy to distinguish the two females in G2 (F12 and F22) and in G4 (F14 and F24) based on their body color, crest shape, and the size of their infants. We could not consistently distinguish the two similar-sized juveniles or the two similar-sized adolescents in G4.

### Distribution pattern of important food species and fruit availability

To investigate plant diversity and forest structure, beginning in December 2007 we established 44 20 × 20 m sample plots throughout the area over which gibbons were known to travel [30]. All plots were located within G1’s home range during later behavioral observation. Due to a dispute over the location of the international border within the study site, we could not establish any plots within G4’s home range. In general,the forest structure and composition in G4′s home range is similar to that of G1.

Plots were placed along six parallel transects that traversed the site, with no fewer than eight plots in each of four distinctive topographical regions (slope, ridgeline, valley, and col) of the site [30]. In July 2010, an additional 29 20 × 20 m plots were set outside any group’s home range in order to survey potential haibitat quality using the same methods. In each plot (N = 73), we measured diameter at breast height (DBH) and tree height for all trees greater than 10 cm diameter (N = 1216). Liana species were also recorded but only in the first 44 plots. Seven food species, each of which contributed > 4.7% and in total 58.4% of their diet based on feeding time [30], were designated important food species. The eighth species contributed 3.1% to the total diet in 2008 and 2009 but contributed much less to the diet (<1%) in 2012 and 2013, therefore, we did not consider it an important species. To characterize the distribution of important foods, we used the species composition from all sample plots to calculated the coefficient of dispersion (CD) for each species, whereby a CD >1 indicates a clumped distribution, a CD <1 a uniform distribution, and a CD = 1 a random distribution [54,55]. If species are distributed randomly, their allocation in the sample should correspond to a Poisson distribution [55]. Therefore, we first tested if the distribution of each important species fit a Poisson distribution using a one-sample Kolmogorov-Smirnov test. We then used the Mann–Whitney *U* test to compare DBH and tree height between each important food species and to all other trees in the plots.

All trees in the first 44 plots, as well as lianas and epiphytes present on these trees, were monitored for seasonal availability of food types (fruit, figs, leaves, buds, and flowers) for three days each month [31]. We estimated the food abundance value (i.e. percent of crown cover) of each food type on a five-point scale, 0–4 (0 < 1%, 1 = 1-25%, 2 = 26-50%, 3 = 51-75%, 4 = 76-100%). The food availability index for non-fig fruit and figs was then calculated based on the food abundance score (0–4) for each fruiting tree, weighted by diameter at breast height (for additional details see [31]). Plant species not eaten by gibbons were not included in calculations of food abundance. Based on natural breaks in the data, months with an availability index of <1200 were designated as lean periods, while months of >2400 were designated as fruiting periods.

### Behavioural observation

From January 2008 to December 2009 (with the exception of February 2008), we spent 7–27 days each month (485 days total), observing gibbon behavior. Whenever possible, groups were observed for the full day. During this period, we observed G1 for 1,455 hours over 215 days from January 2008 to December 2009, G2 for 201 hours over 67 days from August 2008 to December 2009, and G4 for 776 hours over 122 days from August 2008 to December 2009.

Because traditional or standard primate observational techniques (i.e. direct follows) are impossible at this site due to the steepness of the landscape, we observed gibbons from several established observation posts at distances of between 50 and 500 m (most observations were made at a distance of 100–300 m), using 8 × 30 binoculars (Steiner safari) or a spotting scope (Leica Apo-Televid77 20–60) [30,31]. Trees in this site are quite small (on average, tree height was 10.5 m) and gibbons always stayed close to the top of these small trees. Through our Leica telescope we had a good view to observe gibbons even at a distance over 50 m. Our observation posts could cover about 95% of G1’s home range and 85% of G4’s home range. Given the observation conditions focal animal sampling was impractical so we used a scan sampling regime at 5-min intervals to record the activity of gibbons [56]. At each 5-minute point sample, a 1-min scan was made recording the behavior of all visible group members. Each individual was observed for 5 seconds and its predominant behavior was recorded. Inter-individual distance was recorded in an additional 1-min scan between July 2008 and December 2009. We recorded activity as resting, travelling, feeding, grooming, calling, playing, or other [31]. When an individual was feeding, we recorded the food species and specific part eaten (fig, fruit, leaves, buds, flower, invertebrates, and other). Cao vit gibbons usually checked dead leaves and branches before they put something into their mouths and they always moved slowly and at some distance from one another when they were searching for insects, which could be easily distinguished from feeding on other food items (fruit or leaves). We created a map with locations of some emergent trees within each group’s home range on a topographic map superimposed with a 100 × 100 m grid [24]. And then we recorded group locations by referring to these emergent trees and obvious landmarks (rocky outcrops, cliffs, and cols where gibbon groups crossed the ridge). During observations, we recorded all incidents and the context of agonistic behavior (bite, slap, chase, and replacement) among group members *ad libitum*.

### Data analysis

The small sample size of behavioral records in G2 precluded detailed analysis, however, based on our limited feeding records G2 consumed similar food species to G1 and G4. We focused on G1 and G4 in the analysis of feeding competition, activity budget and spatial relationships. We excluded data collected before July when females could not be reliably identified from the analysis of G1. Over the remaining 18 months, we observed G1 on average 65.3 ± 6.5 h (range: 55–81 h, N = 18) on 9 ± 2 days (range: 6–14 d, N = 18) per month. We pooled data for the two juveniles and two adolescents in G4 because we could not reliably distinguish them at all times. In August and December 2008 we observed G4 for only 8 h and 14 h respectively so we excluded data in these two months from the analysis. Over the remaining 15 months between August 2008 and December 2009, we observed G4 on average 50.3 ± 12.6 h (range: 31–68 h, N = 15) on 8 ± 2 days (range: 4–11 d, N = 15) per month.

We calculated the monthly proportional diet and activity budget for each foraging individual (or age/sex group for juveniles and adolescents in G4). We treated monthly diet and activity budgets as independent samples. We compared individual diet and activity budget differences within groups using the Wilcoxon matched pairs test. To eliminate the effect of there being different numbers of immature animals (juveniles and adolescents) in each group, group diet and activity budgets were calculated based on adult group members only. Wilcoxon matched pairs test was used to test for differences in time budget, diet and inter-individual distance between groups after listwise deletion of months for which we did not have data for both groups (N = 15 months for both groups).

To avoid significant underestimation of group spread, we included only those scans in which at least three inter-individual distances were recorded. We used the largest distance to represent group spread during each scan. We then calculated the mean inter-individual distance and group spread for each month by averaging the mean inter-individual distance and maximum inter-individual distance of each scan. These measures were highly correlated in both groups (Spearman correlation: G1, r = 0.984, P < 0.001; G4, r = 0.994, P < 0.001). Because G4 consisted of more independent individuals, we further calculated each index using scans in which six inter-individual distances were recorded; however, both inter-individual distance (Spearman correlation: r = 0.932, P < 0.00) and group spread (Spearman correlation: r = 0.915, P < 0.001) were highly correlated using three or six distance samples. Therefore, only results based on three inter-individual distances are reported for each group.

Because of fog, rain and the difficult terrain, full-day observations (i.e. visually tracking animals from sleeping tree to sleeping tree) were difficult to achieve [31]. In order to increase sample size, days in which gibbons were lost for less than one hour (one or two group locations missed) were also used to estimate daily path length (DPL; the sum of all distances between consecutive group locations for one day). We measured DPL using software ImageJ [57]. In total, we had 59 DPLs for G1 between June 2008 and December 2009, and 30 DPLs for G4 between October 2008 and December 2009. To control for seasonal variation in DPL, we only used those collected during the same period (between October 2008 and December 2009) to compare differences in DPL between groups (G1: 50 days; G4: 30 days) using Mann–Whitney *U* test.

### Ethical standards

The study was conducted in Bangliang Nature Reserve, Jingxi County, Guangxi, China. All research reported in this manuscript has met the appropriate national and institutional guidelines for the legal acquisition and was permitted by the Guangxi Forestry Bureau and Bangliang Nature Reserve.

## References

[1] Cody ML (1971). Finch flocks in the Mohave Desert. Theor Popul Biol.

[2] Hamilton WD (1971). Geometry for the selfish herd. J Theor Biol.

[3] Wrangham RW (1980). An ecological model of female-bonded primate groups. Behaviour.

[4] van Schaik CP, Dunbar RIM (1990). The evolution of monogamy in large primates: a new hypothesis and some crucial tests. Behaviour.

[5] Chapman CA, Chapman LJ, Boinski S, Garber P (2000). Determinants of group size in social primates: the importance of travel costs. On the move: how and why animals travel in groups.

[6] Nicholson AJ (1954). An outline of the dynamics of animal populations. Aust J Zool.

[7] Sterck EHM, Watts DP, van Schaik CP (1997). The evolution of female social relationships in nonhuman primates. Behav Ecol Sociobiol.

[8] Koenig A (2002). Competition for resources and its behavioral consequences among female primates. Int J Primatol.

[9] Schulke O (2003). To breed or not to breed–food competition and other factors involved in female breeding decisions in the pair-living nocturnal for-marked lemur (Phaner furcifer). Behav Ecol Sociobiol.

[10] Borries C, Larney E, Lu A, Ossi K, Koenig A (2008). Costs of group size: lower developmental and reproductive rates in larger groups of leaf monkeys. Behav Ecol.

[11] Savini T, Boesch C, Reichard UH (2009). Varying ecological quality influences the probability of polyandry in white-handed gibbons (*Hylobates lar*). Biotropica.

[12] Reichard UH, Ganpanakngan M, Barelli C, Kappeler PM, Watts DP (2011). White-handed gibbons of Khao Yai: social flexibility, complex reproductive strategies, and a slow life history. Long-term field studies of primates.

[13] Lappan S (2007). Social relationships among males in multimale siamang groups. Int J Primatol.

[14] Haimoff EH, Yang XJ, He XJ, Chen N (1986). Census and survey of wild black-crested gibbons (*Hylobates concolor*) in Yunnan Province, People’s Republic of China. Folia Primatol.

[15] Brockelman W, Lappan S, Whittaker DJ (2009). Ecology and the social system of gibbons. The gibbons: new perspectives on small ape socioecology and population biology.

[16] Brockelman WY, Nathalang A, Greenberg DB, Suwanvecho U, Yamagiwa J, Karczwaski L (2014). Evolution of small-group territoriality in gibbons. Primates and cetaceans: field studies and conservation of complex mammalian societies.

[17] Leighton DR: G, Smuts BB, Cheney DL, Seyfarth RM, Wrangham RW, Struhsaker TT (1987). Territoriality and monogamy. Primate Societies.

[18] Bartlett TQ (2009). The gibbons of Khao Yai: seasonal variation in behavior and ecology.

[19] Liu ZH, Zhang YZ, Jiang HS, Southwick C (1989). Population structure of *Hylobates concolor* in Bawanglin Nature Reserve, Hainan, China. Am J Primatol.

[20] Zhou J, Wei FW, Li M, Chan BPL, Wang DL (2008). Reproductive characters and mating behaviour of wild *Nomascus hainanus*. Int J Primatol.

[21] Fan PF, Jiang XL, Liu CM, Luo WS (2006). Polygynous mating system and behavioural reason of black crested gibbon (*Nomascus concolor jingdongensis*) at Dazhaizi, Mt. Wuliang, Yunnan, China. Zool Res.

[22] Fan PF, Jiang XL (2010). Maintenance of multifemale social organization in a group of *Nomascus concolor* at Wuliang Mountain, Yunnan, China. Int J Primatol.

[23] Huang B, Guan ZH, Ni QY, Orkin JD, Fan PF, Jiang XL (2013). Observation of intra- and extra-group copulation and reproductive characters in free ranging groups of western black crested gibbon (*Nomascus concolor jingdongensis*). Inter Zool.

[24] Fan PF, Fei HL, Xiang ZF, Zhang W, Ma CY, Huang T (2010). Social structure and group dynamics of the cao vit gibbon (*Nomascus nasutus*) in Bangliang, Jingxi, China. Folia Primatol.

[25] Srikosamatara S, Brockelman WY (1987). Polygyny in a group of pileated gibbons via a familial route. Int J Primatol.

[26] Ahsan F (1995). Fighting between two females for a male in the Hoolock gibbon. Int J Primato.

[27] Sommer V, Reichard U, Kappeler PM (2000). Rethinking monogamy: the gibbon case. Primate males: cause and consequences of variation in group composition.

[28] Guan ZH, Huang B, Ning WH, Ni QY, Sun GZ, Jiang XL (2013). Significance of grooming behavior in two polygynous groups of western black crested gibbons: implication for understanding social relationships among immigrant and resident group members. Am J Primatol.

[29] Brockelman WY, Reichard U, Treesucon U, Raemaekers J (1998). Dispersal, pair formation and social structure in gibbons (*Hylobates lar*). Behav Ecol Sociobiol.

[30] Fan PF, Fei HL, Scott MB, Zhang W, Ma CY (2011). Habitat and food choice of the critically endangered cao vit gibbon (*Nomascus nasutus*) in China: Implications for conservation. Biol Conserv.

[31] Fan PF, Fei HL, Ma CY (2012). Behavioral responses of cao vit gibbon (*Nomascus nasutus*) to variations in food abundance and temperature in Bangliang, Jingxi, China. Am J Primatol.

[32] Mootnick A, Mootnick AR, Fan PF (2011). A comparative study of crested gibbons (*Nomascus*). Am J Primatol.

[33] Zhou QH, Wei FW, Li M, Huang CM, Luo B (2006). Diet and food choice of *Trachypithecus francoisi* in the Nonggang Nature Reserve, China. Int J Primatol.

[34] Huang CM, Wu H, Zhou QH, Li YB, Cai XW (2007). Feeding strategy of Francois’ langur and white-headed langur at Fusui, China. Am J Primatol.

[35] Workman C (2010). Diet of the Delacour’s langur (*Trachypithecus delacour*) in Van Long Nature Reserve, Vietnam. Am J Primatol.

[36] Wheeler BC, Scarry CJ, Koenig A (2013). Rates of agonism among female primates: a cross-taxon perspective. Behav Ecol.

[37] Chivers DJ (1974). The siamang in Malaya: a field study of a primate in a tropical forest. Contr Primat.

[38] Bartlett TQ, Campbell CJ, Fuentes A, MacKinnon KC, Stumpf RM, Bearder SK (2011). The Hylobatidae: small apes of Asia. Primates in perspective.

[39] Gittins SP (1982). Feeding and ranging in the agile gibbon. Folia Primatol.

[40] McConkey KR, Ario A, Aldy F, Chivers DJ (2003). Influence of forest seasonality on gibbon food choice in the rain forests of Barito Ulu, Central Kalimantan. Int J Primatol.

[41] Kim S, Lappan S, Choe J (2011). Diet and ranging behavior of the endangered Javan gibbon (*Hylobates moloch*) in a submontane tropical rainforest. Am J Primatol.

[42] Reichard UH, Barelli C (2008). Life history and reproductive strategies of Khao Yai Hylobates lar: Implications for social evolution in apes. Int J Primatol.

[43] Chivers DJ, Raemaekers JJ, Chivers DJ (1980). Long-term changes in behaviour. Malayan forest primates: Ten years’ study in tropical rain forest.

[44] Palombit RA (1995). Longitudinal patterns of reproduction in wild female Siamang (*Hylobates syndactylus*) and white-handed gibbons (*Hylobates lar*). Int J Primatol.

[45] Mitani JC (1990). Demography of agile gibbons (*Hylobates agilis*). Int J Primatol.

[46] Lappan S (2008). Male care of infants in a siamang (*Symphalangus syndactylus*) population including socially monogamous and polyandrous groups. Behav Ecol Sociobiol.

[47] Geissmann T. Evolution of Communication in Gibbons (Hylobatidae). Ph.D. dissertation. Universitaet Zuerich, Zuerich; 1993.

[48] Zhao QK (1994). Seasonal change in body weight of *Macaca thibetana* at Mt. Emei, China. Am J Primatol.

[49] Knott CD (1998). Changes in orangutan caloric intake, energy balance, and ketones in response to fluctuating fruit availability. Int J Primatol.

[50] Ptak SE, Lachmann M (2003). On the evolution of polygyny: a theoretical examination of the polygyny threshold model. Behav Ecol.

[51] Fan PF, Ren GP, Wang W, Scott MB, Ma CY, Fei HL (2013). Habitat evaluation and population viability analysis of the last popualtion of cao vit gibbon (*Nomascus nasutus*): implications for conservation. Biol Conserv.

[52] La QT, Trinh DH, Long B, Geissmann T: Status review of black crested gibbons (Nomascus concolor and Nomascus sp. cf. nasutus) in Vietnam [abstract]. In Caring for primates, Abstracts of the XIXth congress of the International Primatological Society, 4th- 9th August, 2002, Beijing, China. 2002: 131–32.

[53] Chan BPL, Tan XF, Tan WJ (2008). Rediscovery of the Critically Endangered eastern black-crested gibbon *Nomascus nasutus* (Hylobatidae) in China, with preliminary notes on population size, ecology and conservation status. Asian Primates J.

[54] Koenig A, Beise J, Chalise MK, Ganzhorn JU (1998). When female should contest for food-testing hypotheses about resource density, distribution, size and quality with Hanuman langurs (*Presbytis entellus*). Behav Ecol Sociobiol.

[55] Saj TL, Sicotte P (2007). Predicting the competitive regime of female Colobus vellerosus from the distribution of food resources. Int J Primatol.

[56] Altmann J (1974). Observational study of behaviour: sampling methods. Behaviour.

[57] Abramoff MD, Magalhaes PJ, Ram SJ (1995). Image processing with ImageJ. Biophotonics Int.

